# Surface Electromyography of the Vastus Lateralis, Biceps Femoris, and Gluteus Medius in Dogs During Stance, Walking, Trotting, and Selected Therapeutic Exercises

**DOI:** 10.3389/fvets.2019.00211

**Published:** 2019-07-10

**Authors:** Hannah McLean, Darryl Millis, David Levine

**Affiliations:** ^1^Department of Small Animal Clinical Sciences, University of Tennessee College of Veterinary Medicine, Knoxville, TN, United States; ^2^Department of Physical Therapy, University of Tennessee at Chattanooga, Chattanooga, TN, United States

**Keywords:** surface electromyography, canine, rehabilitation, exercise, physical therapy

## Abstract

**Objective:** The objective of the study reported here was to evaluate the muscle activity patterns of the vastus lateralis (VL), biceps femoris (BF), and gluteus medius (GM) during stance, walking, trotting, and selected therapeutic exercises in clinically sound, healthy dogs. Our hypothesis was that the muscle activity during all exercises would differ from muscle activity at the stance.

**Methods:** Surface electromyography of the selected muscles was performed during stance, walking, trotting, elevation of forelimbs on a platform, elevation of forelimbs on a platform with hindlimbs on an inflatable balance device, stepping up onto and over an obstacle, standing on a wobble board, dancing backwards, and wearing a leg weight at the walk and the trot. The maximal and mean muscle amplitude (μV) reflecting activity during several motion cycles were compared among the exercises.

**Results:** Mean EMG amplitude of the BF was significantly higher in all exercises (*p* < 0.05) in comparison to stance. Mean EMG amplitude of the VL was significantly higher (*p* < 0.05) during walking, trotting, dancing backwards, stepping up and over an obstacle, and at a trot with a leg weight as compared to stance. Mean EMG amplitude of the GM was significantly higher (*p* < 0.05) during trotting, at a walk and a trot with a leg weight, standing on a wobble board, stepping up and over an obstacle, and dancing backwards when compared to stance. Of the studied exercises, dancing backwards increased the mean EMG amplitude of the BF and GM to the largest degree. Stepping up and over an obstacle increased the mean EMG amplitude of the VL to the largest degree.

**Conclusion:** Compared to stance, the majority of therapeutic exercises examined increased muscle activity to varying degrees in the BF, VL, and GM. Our results may help clinicians to choose specific exercises to target specific muscles during conditioning, strengthening and rehabilitation.

## Introduction

Therapeutic exercises are an essential part of rehabilitation of musculoskeletal and neurologic injuries of veterinary patients. Therapeutic exercises to improve active joint motion, and to build strength, power and speed are used to help return the patient to as normal a function as possible (1, 2). When designing therapeutic exercise programs for veterinary patients, multiple factors should be considered including the diagnosis, affected tissues and their stage of healing, pre-morbid and co-morbid conditions, available range of motion in the joints exercised, and overall health of the patient including cardiovascular status (1–3). To fully develop a specific therapeutic exercise program, knowledge of muscle activity patterns and joint biomechanics during various exercises is necessary. In human medicine, surface electromyography has been used extensively to study muscle roles and interactions in healthy and pathologic states. A recent study published by Chen et al. (4) evaluated activations of the vastus lateralis, vastus medialis oblique and the gluteus medius muscles in females during hip abduction and external rotator movements, and open and closed kinetic chain knee extension movements. This study suggested that selective gluteus medius muscle activation was induced during hip abduction and external rotation movements, accompanied by an increase in vastus lateralis muscle activation. It also revealed that in open and closed kinetic chain knee movements, the ratio of the vastus lateralis to the vastus medialis oblique muscle activity approached 1:1. More selective vastus medialis oblique muscle activation was induced during the closed kinetic chain knee movement (4). While the degree of specificity of exercise prescription in veterinary medicine is not as developed as in human medicine, it is expanding, in part, due to studies using electromyography.

Several studies have evaluated canine muscle activity using needle or surface electromyography (sEMG) (5–11) while walking, trotting, and during other exercises. One study compared the activity patterns of the vastus lateralis, biceps femoris, and gluteus medius in sound dogs during over ground walking, walking on a treadmill at an 11% incline or decline, and walking over cavaletti rails (10). This study concluded that cavaletti and incline walking exercises significantly increased vastus lateralis and gluteus medius activity compared to over ground walking (especially during the early swing phase) (10). Another study evaluated the EMG activity of 4 forelimb muscles (biceps brachii, supraspinatus, infraspinatus, and the long head of the triceps brachii) in border collies during two specific agility tasks (jumping and A-frame). This study concluded that all of the muscles were activated at higher levels compared to walking (1.7–10.6 times based on the activity) and that jumping is an especially demanding activity for dogs (7). These studies allow the veterinary practitioner a better understanding of the pattern of muscle activation during these specific tasks and allows for a better understanding of the potential causes of injuries.

The objective of the study reported here was to analyze the mean and maximum muscle activation patterns of the vastus lateralis (VL), biceps femoris (BF), and gluteus medius (GM) during stance, walking, trotting and specific therapeutic exercises in clinically sound, healthy dogs. Our hypothesis was that the muscle activity during all therapeutic exercises would differ from the muscle activity at the stance. Our overall goal was to help guide clinicians in the selection of specific exercises to selectively strengthen the BF, VL, and GM in dogs during physical rehabilitation.

## Materials and Methods

### Dogs

Privately owned and clinically sound dogs (*n* = 12; 2 Standard Poodles, 1 Golden Retriever, 1 Australian shepherd, and 9 Mixed Breed Dogs) were enrolled in the study with written owner consent. Dog ages and weights ranged from 1 to 9 years (mean 3.8 years) and 17–33.1 kg (mean 25.6 kg), respectively. Dogs were only included in the study if they had a body condition score of 4 or 5 on a scale of 9. Five dogs were female and seven were male. All dogs were spayed or neutered. All dogs had a complete neurologic and orthopedic examination prior to enrollment; dogs were excluded if they showed any signs of visible lameness or pain upon palpation of the joints, spine, or skeletal muscles or if a gait abnormality at the walk or trot, posture abnormality, or any other orthopedic or neurologic conditions were detected. In order to confirm that dogs had no lameness, kinetic data were obtained using a force platform (AMTI, Watertown, MA). Four valid trials for each side of the dog were obtained at a trot. For a trial to be considered valid, dogs must have no sudden deviation of gait, sudden head movements, turning of the head during gait, or any other motion that might affect collection of kinetic data. Velocity and acceleration of the dog and handler were maintained between 1.7 and 2.1 m/s and ± 0.40 m/s^2^, respectively, using five photocells and a start-interrupt timer system. Mean peak vertical force values were used to identify weight-bearing asymmetry for each dog; dogs were excluded from the study if there was >5% asymmetry of the forelimbs or hindlimbs. No dogs were excluded based on kinetic analysis criteria. This study was approved by the University of Tennessee Institutional Animal Care and Use Committee and was performed in accordance with AAALAC and USDA guidelines.

### sEMG

**Figure 1:**
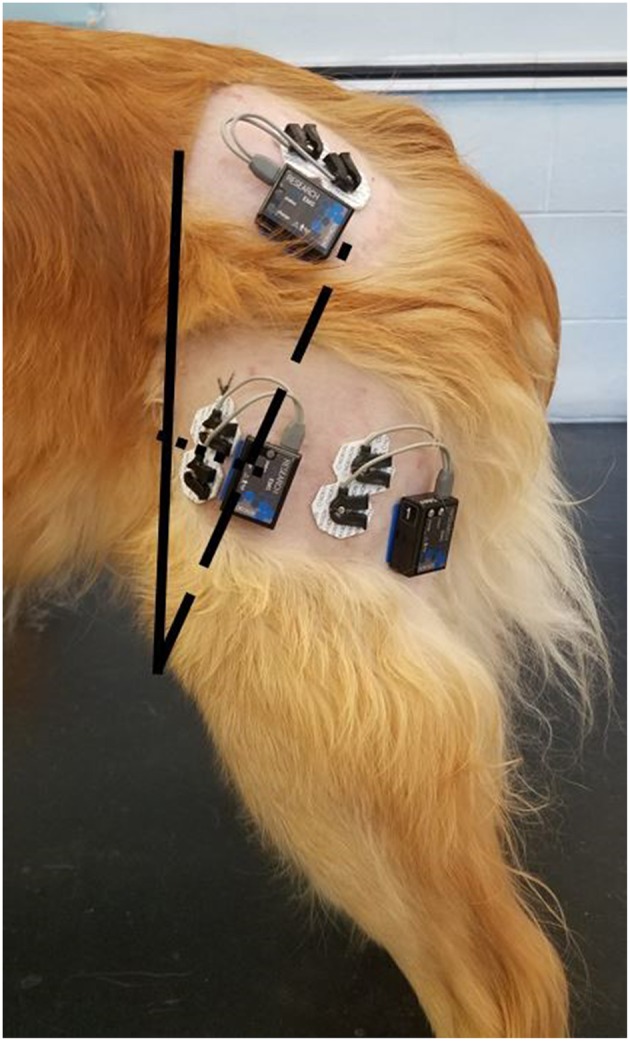
Placement of the surface electrodes over the vastus lateralis, biceps femoris, and gluteus medius. For the vastus lateralis, the solid line represents the distance between the iliac crest and the proximal aspect of the patella; the dashed line represents the distance between the greater trochanter and the proximal aspect of the patella; the dotted line represents the junction between the middle of the dashed and solid lines.

Muscle potentials were recorded using a telemetric unit (Myomotion; Noraxon USA, Inc., Scottsdale, AZ). Self-adhesive dual surface electrodes (4 cm × 2.2 cm) with an inter-electrode distance of 2 cm were used. The electrodes were positioned and attached to the shaved, clean skin of the left pelvic limb, and the muscle activities of the VL, the cranial part of the BF, and the GM were evaluated using the same methodology as previously described (8–11). For each muscle, the dual electrodes were placed with the dog in a standing position and the feet positioned squarely underneath the body. The same examiner placed the electrodes on each muscle to ensure consistent positioning. For the BF muscle, the electrodes were placed in the middle third of the distance between the ischial tuberosity and patella. For the VL muscle, the distance between the iliac crest and patella and between the patella and greater trochanter were measured. The central points of these lines were connected, and the electrodes were placed in the center of the resulting line (Figure 1). For the GM muscle, the electrodes were placed at the midpoint of the distance between the iliac crest and greater trochanter.

### sEMG Acquisition Procedure

Surface electromyography was performed during stance, walking, trotting, and during different therapeutic exercises. Before the testing sessions were recorded, each dog was allowed to walk and trot through the testing area with the sensors attached until they were visibly comfortable and no obvious gait asymmetry was present as a result of instrumentation. Surface EMG activity, measured in microvolts, was recorded simultaneously for all muscle groups while the dogs performed each of the exercises, and all exercises were performed during the same testing period so that the electrodes were not moved during data collection.

The measurement for stance was taken with the dog standing squarely in the testing area. The remaining exercises were divided into static and dynamic exercises. All exercises were performed in the same order, with a 2 min break between exercises to allow for rebuilding of the testing area between each exercise.

**Figure 2:**
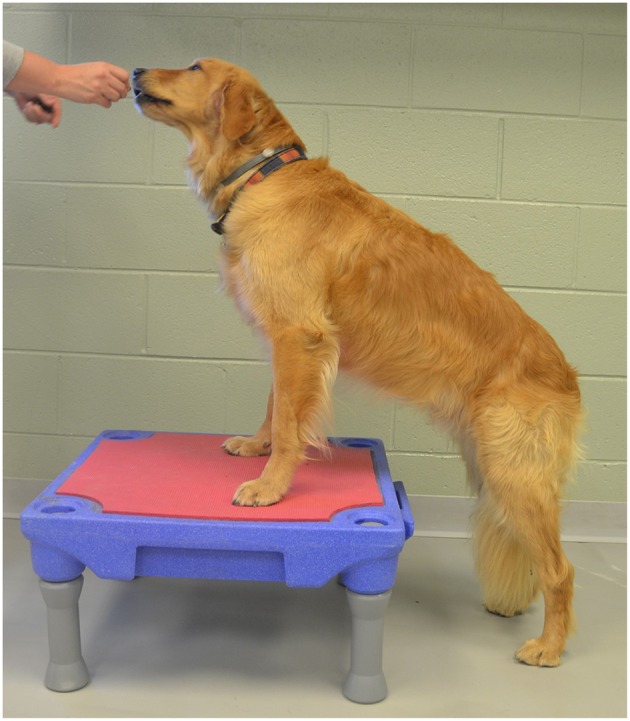
Elevation of the forelimbs onto a 30 cm tall platform.

**Figure 3:**
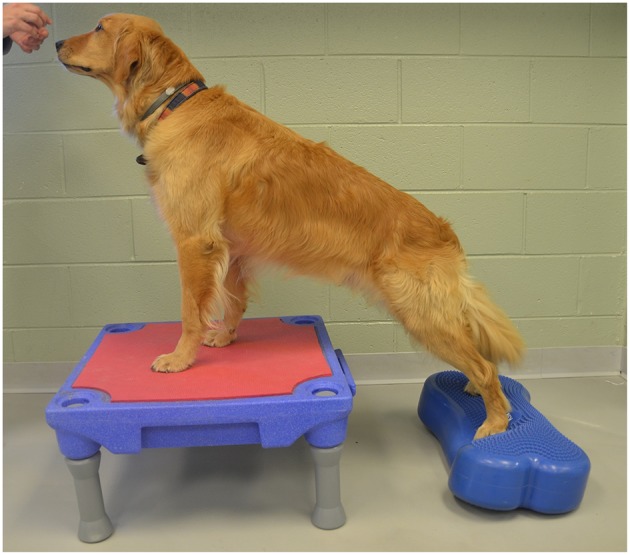
Elevation of the forelimbs onto the same 30 cm platform with the hindlimbs on a 15 cm tall inflatable balance device.

**Figure 4:**
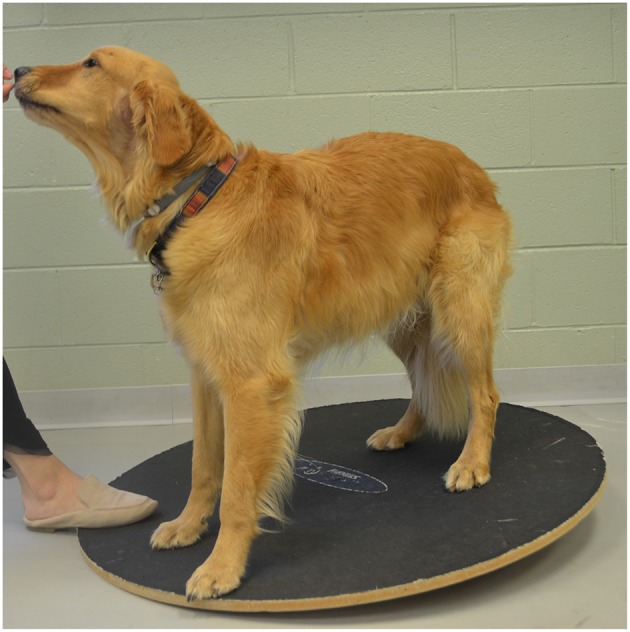
Standing on a round 91 cm balance board.

The static exercises included:

1. Elevation of the forelimbs onto a 30 cm tall platform (Sports Klimb, FitPAWS, Longmont, CO) (Figure 2).
2. Elevation of the forelimbs onto the same 30 cm platform with the hindlimbs on a 15 cm tall inflatable balance device (CanineGym® K9FITbone, FitPAWS, Longmont, CO) (Figure 3).
3. Standing on a round 91 cm balance board (FitPAWS® Wobble Board, FitPAWS, Longmont, CO) (Figure 4).

**Figure 5:**
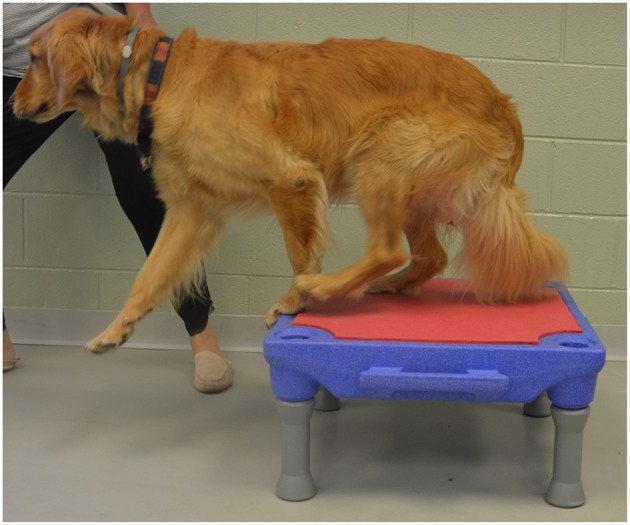
Up and over.

**Figure 6:**
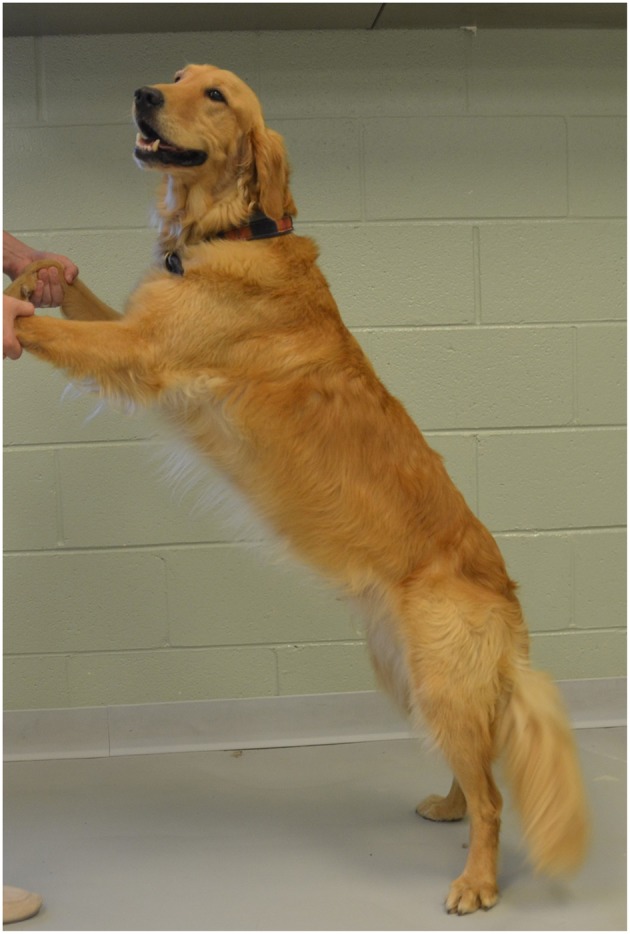
Backwards dancing.

Dynamic exercises included:

1. Walking at a comfortable speed for three trials of 7–10 strides.
2. Trotting at a comfortable speed for three trials of 7–10 strides.
3. “Up and over” which required each dog to walk up onto a 30 cm tall platform (Sports Klimb, FitPAWS, Longmont, CO) and immediately walk down the other side (Figure 5).
4. Backwards dancing (Figure 6).

After these exercises were finished the testing area was cleared and a 0.27 kg (0.6 lb) limb weight (Weight Adjustable Hands Free® Wrist Weights, All Pro Exercise Products, Hillsborough, NJ) was applied just proximal to the hock. The dogs were allowed to become accustomed to the weight for 2–3 min, and were then walked and trotted at a comfortable speed for three trials of 7–10 strides.

### Data Processing

As previously described, the raw EMG data (in microvolts) were full-wave rectified and filtered using a low pass filter at 4 Hz (8, 10). The raw data were then smoothed using a root mean square envelope of 100 ms. The mean and maximal amplitude of each exercise were then calculated. For static exercises, 10 s of continuous raw EMG data was analyzed. For dynamic exercises, 3 strides of the left hindlimb were analyzed.

### Statistical Analysis

Data were analyzed using mixed model analysis, with the mean and maximum as the response variables, exercise and muscle as the fixed effects, and subject as the random effect. Diagnostic analysis was performed on residuals and ranked transformation was applied if non-normality and unequal variance were present. *Post-hoc* multiple comparisons for fixed effects were conducted with Tukey's adjustment. Statistical significance was identified at a significance level of 0.05. All analysis was conducted using PROC MIXED in SAS 9.4 TS1M3 from SAS Institute Inc. (Cary, NC).

## Results

### Mean Amplitude

**Figure 7:**
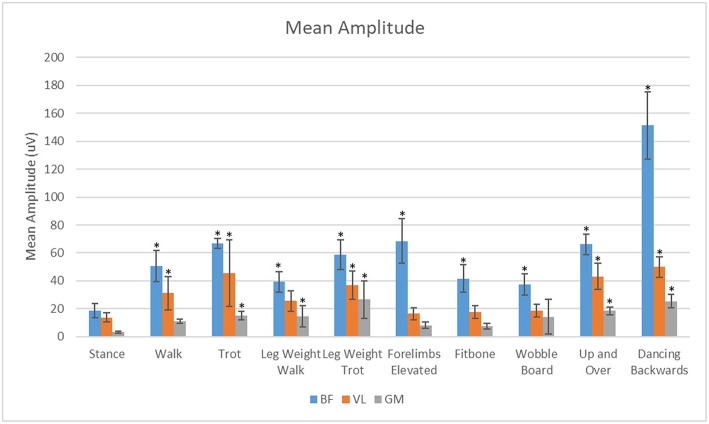
Analysis of the effects of exercise on mean sEMG amplitude of the biceps femoris (BF), gluteus medius (GM), and vastus lateralis (VL) muscles. *Significant difference from stance (*p* < 0.05).

For all exercises evaluated in this study, the BF had significantly greater (*p* < 0.05) mean amplitude than the VL or the GM (Figure 7). The VL also had significantly greater (*P* < 0.05) mean amplitude than the GM in all exercises evaluated.

### Maximal Amplitude

**Figure 8:**
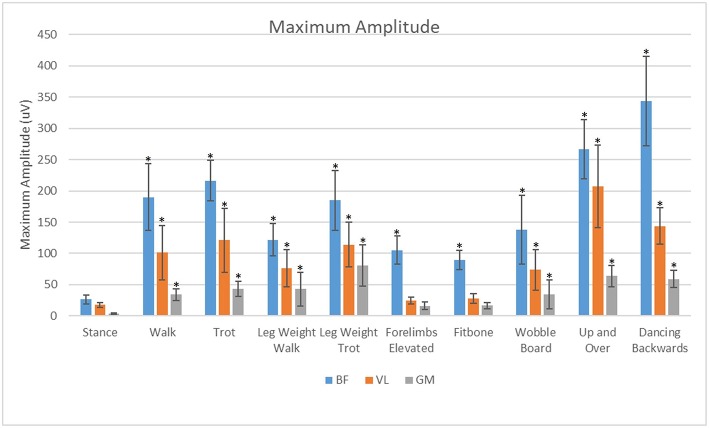
Analysis of effects of exercise and muscle on maximum amplitude of the biceps femoris (BF), gluteus medius (GM), and vastus lateralis (VL). *Significant difference from stance (*p* < 0.05).

For all exercises evaluated in this study, the BF had significantly greater (*p* < 0.05) maximal sEMG amplitude than the VL or the GM (Figure 8). The VL had significantly greater (*P* < 0.05) maximal sEMG amplitude than the GM in all exercises.

## Discussion

Our main purpose was to evaluate the muscle activity patterns of the VL, BF, and GM during stance, walking, trotting and specific therapeutic exercises in clinically sound, healthy dogs. We chose to evaluate the BF, VL and GM muscles because they are major stabilizers of the hip and stifle, and they are large and superficial enough to have reasonable expectations of obtaining sEMG activity (12, 13). These muscles also tend to atrophy with orthopedic disease or injury (14). In this study each exercise and muscle had similar trends between maximal amplitude and mean amplitude. In theory, therapeutic exercises could have a brief high peak vs. a low average while other exercises could have a longer sustained contraction with a lower peak. At this time, it is unknown whether longer sustained contractions or brief periods of maximal amplitude are more important for strengthening and function. Our hypothesis that the muscle activity during all therapeutic exercises would differ from the muscle activity at a stance was supported for all exercises in the BF for both mean and maximal sEMG activity. The mean amplitude of the VL was not significantly different between stance and walking with the leg weight or in the static exercises. The mean amplitude of the GM was not significantly different between stance and walking or with the static exercises.

The biceps femoris was most activated during the dynamic exercises such as dancing backwards and the up and over although it was significantly elevated over stance in both mean and maximal amplitude for all exercises. It was our goal to measure primarily the cranial portion of the biceps femoris responsible for hip and stifle extension. It is possible that the increased response of the biceps femoris in the dynamic exercises may be attributed, in part, to additive effects and cross talk. This cross talk or sum-signal of nearby muscles could be from the caudal portion of the biceps femoris responsible for stifle flexion, which may increase the mean amplitude because of continual activation throughout the gait cycle during dynamic exercises. It is also conceivable that we could have cross-talk from the semitendinosus or semimembranosus muscles, further contributing to this signal.

The vastus lateralis was maximally activated during the up and over and dancing exercises. This may be due to the increased load placed on this muscle to do the work of pushing the body weight a vertical distance in the up and over exercise, which is similar to stair ascent (3). The vastus may have been maximally activated by the dancing backwards exercise because of the additional force placed on just the two pelvic limbs, and possibly because of a sustained eccentric contraction of the quadriceps muscle with increased stifle flexion which occurs with dancing backward as compared with normal walking or dancing forward.

The gluteus medius was most active during the leg weight trotting and backwards dancing exercises. We theorize that this may be due to the increased hip extension and dynamic movement during these exercises. During trotting, there is a 5° increase in hip extension and during dancing backwards there is a 28° increase in hip extension compared with walking (3). The mean amplitude of the gluteus medius was lowest in the static exercises.

The diminished mean and maximal activation of all three muscles groups with the addition of the leg weight at the walk was an unexpected finding. We postulate that this may be due to the limited amount of weight applied, activation of other muscle groups to assist with advancing the limb with added weight, or due to a change in gait with the addition of the leg weight. Although all dogs were allowed a short time to acclimate to the leg weight, there was still an observable subjective change in their gait. This subjective change was characterized by a shortened stride length, diminished stance time and an increased postural sway to the unweighted hind limb. A small unpublished kinetic study performed by the investigators revealed a diminished vertical impulse and peak vertical force in hindlimbs with the same weight applied. It was observed that the dogs became asymmetric and applied more force to the opposite hindlimb and the contralateral forelimb in order to compensate for the diminished weight bearing on the limb with the leg weight applied. It is possible that additional time for acclimation may have reduced or eliminated the gait asymmetry. However, in a clinical situation, it may not be possible to devote long periods of time for acclimation. Therefore, we believe that our results likely mimic the clinical situation.

Another consideration in translation of our data to clinical use is that electromyography is often used to infer the pattern of production of force by skeletal muscle. According to Roberts et al., the interpretation of muscle function from the EMG is challenged by the fact that factors such as type of muscle fiber, muscle length, and force-velocity curve can all influence the relationship between electrical and mechanical activity of a muscle (15) In his study, Roberts evaluated muscles of wild turkeys with simultaneous measurements of EMG, muscle force, and fascicle length in hindlimb muscles. This allowed them to probe the quantitative link between muscle contraction force and EMG. They concluded that during stance, force amplitude was linearly related to mean EMG amplitude but that forces during swing phase were lower than predicted from the stance phase force–EMG relationship. Together the results suggest that any inference of force from EMG must be done cautiously when a broad range of activities is considered.

Although our results provide significant information to advance our knowledge of surface EMG in dogs performing specific exercises, there are several limitations to this study. Results of surface EMG measurements can be affected by multiple factors. A surface EMG represents a sum signal of the target and nearby muscle activities which can result in falsely reported muscle activity. This phenomenon is termed cross-talk. Cross-talk may have been limited with the use of ultrasound guided fine wire EMG. We elected surface EMG due to the less invasive nature and reported similarities between fine needle and surface electromyography in the human vastus medialis and biceps femoris muscles (16). Another challenge of EMG recording in dogs is that the skin is freely movable and the electrodes may have moved over other muscle bellies during the exercises. Body fat content has also been shown to influence sEMG measurements (17). In this study we attempted to minimize this effect by working with dogs of similar size and body condition score. Based on the symmetry of ground reaction forces in the pelvic limbs of each dog, it was assumed that muscle activity would be symmetrical in both pelvic limbs, but this assumption has not yet been proven. Although the instrumented limb may have had different muscle activation compared with the contralateral limb, we believe this effect was minimal. However, it is possible that our results might have been influenced by the fact that we tested only the left pelvic limb.

The goal of this study was to build upon previous research in order to begin to catalog the timing and magnitude of muscle amplitude for many different muscles powering a variety of types of locomotion. In humans, these amplitudes in normal and abnormal movement have been elucidated allowing for a better understanding of muscle activation during exercise thus allowing targeted strengthening and conditioning protocols to be prescribed. The specific results of this study aim to help the practitioner build upon previous work by Bockstahler et al. (8) and Breitfuss et al. (10) but include many additional exercises as well as a larger sample size. The muscle amplitude during the new exercises described in this study is novel research which has not yet been published. This may help the practitioner determine which exercises could help with strengthening and conditioning of the specific muscles in the study. The muscles described in this study were chosen due to their specific atrophy during both acute and chronic conditions of the coxofemoral and stifle joints such as cranial cruciate ligament injury and osteoarthritis. A 2006 study by Monk (18) revealed that there was significantly reduced thigh circumference in cranial cruciate deficit limbs prior to surgery but that early intensive post-operative therapy can help prevent continued muscle atrophy and build muscle mass and strength. The results of this study can additionally provide the practitioner with specific knowledge of muscles. For example, to specifically target the biceps femoris, adding additional weight to the affected limb results in muscle amplitude. This weight may be in the form of body weight training such as elevating the forelimbs (as with the dancing and forelimbs elevated exercise) or by increasing the force on the muscle by increasing velocity (such as at the trot or trotting with a leg weight).

Despite these challenges, our study provides new insights and basic data on the muscle activity of the pelvic limbs during various exercises as compared to a stance. Mean EMG amplitude of the BF was increased in all exercises in comparison to stance with the largest increase observed during the dancing exercise and elevation of forelimbs. Mean EMG amplitude of the VL was greatest during dancing, and stepping up and over an obstacle. Mean EMG amplitude of the GM was increased primarily during dancing, up and over, and at the trot with a leg weight. Our results should be considered when deciding on a specific exercise to strengthen the BF, VL, or GM muscles in an individual canine rehabilitation program.

## Ethics Statement

This study was carried out in accordance with the recommendations of the institutional animal care and use committee. The protocol was approved by the institutional animal care and use committee.

## Author Contributions

We certify that all authors meet the qualifications for authorship as listed below: (1) substantial contributions to the conception or design of the work or the acquisition, analysis, or interpretation of data for the work, (2) drafting the work or revising it critically for important intellectual content, (3) final approval of the version to be published, (4) agreement to be accountable for all aspects of the work in ensuring that questions related to the accuracy or integrity of any part of the work are appropriately investigated and resolved.

### Conflict of Interest Statement

The authors declare that the research was conducted in the absence of any commercial or financial relationships that could be construed as a potential conflict of interest.
